# Force–velocity profile during vertical jump cannot be assessed using only bodyweight jump and isometric maximal voluntary contraction tasks

**DOI:** 10.1038/s41598-020-76262-4

**Published:** 2020-11-05

**Authors:** Nejc Šarabon, Žiga Kozinc, Goran Marković

**Affiliations:** 1 Faculty of Health Sciences, University of Primorska, Polje 42, 6310 Izola, Slovenia; 2 Laboratory for Motor Control and Motor Behavior, S2P, Science To Practice, Ltd, Tehnološki Park 19, 1000 Ljubljana, Slovenia; 3 Andrej Marušič Institute, University of Primorska, Muzejski trg 2, 6000 Koper, Slovenia; 4 Faculty of Kinesiology, University of Zagreb, Horvaćanski zavoj 15, 10110 Zagreb, Croatia; 5 Motus Melior Ltd, Hektorovićeva ul. 2, 10000 Zagreb, Croatia

**Keywords:** Physiology, Medical research

## Abstract

Recently, the two-point method of force–velocity (F–V) profiling of multi-joint human movements has been introduced and validated. In this study, we investigated the validity of estimating the jumping F–V profile using only bodyweight jump and isometric maximal voluntary contraction (MVC) task. Participants (n = 30) performed 3 repetitions of squat (SJ) and counter-movement jumps (CMJ), each at loads that were progressively increased by 10 kg increments, with the number of loads depending on the individual’s ability. Then, 3 isometric MVC trials were performed in 3 knee angles (30°, 60° and 90°). F–V profiling of SJ and CMJ were performed using the multiple-point method, the two-point method, and the novel Jump-MVC method. The results showed poor to fair validity of the novel Jump-MVC method for assessing jumping F–V profile (most ICC < 0.5, most CV > 10%, significant systematic bias present, and the presence of proportional bias). The exception was the estimation of theoretical maximal power, which was highly valid for both SJ and CMJ (ICC = 0.91–0.95; CV = 5.0–6.3%). In contrast, validity of the two-point method was excellent (all ICC > 0.90; CV = 2–6%). Although additional studies are needed, present results suggest that the F–V profiling of vertical jumps should be performed using the two-point method with distal loads.

## Introduction

It is well documented that vertical jumping performance in athletes is associated with athletic performance^1–5^, notably sprinting^1–3^ and change-of-direction ability^1,4,5^. Parameters associated with vertical jump performance have been shown to be highly reliable^6–10^. While force plates are considered a gold standard for vertical jump testing, lower-cost alternatives, such as certain jumping (i.e. contact) mats^11–13^ also provide trustworthy estimation of jump height. Although jump height, along with force- and power-related parameters assessed during vertical jumps are associated with athletic performance, these variables represent only a limited aspect of jumping ability and cannot distinguish between force-, velocity- and power producing capacity^14,15^. For this reason, there has recently been an increased interest in exploring the individual’s force–velocity (F–V) profile^16–21^.

Inverse linear relationship between force/torque and linear/angular velocity has been documented for several multi-joint movements^22^. It has been shown that performing resistance exercise can alter the F–V profile^19,23^ and that these changes in F–V profile depend on the type of resistance exercise^19^. It was also shown that athletes of different sports are characterized by substantially different F–V profiles^24^. Furthermore, theoretical simulations have shown that an optimal F–V profile (i.e. the slope of the inverse linear F–V relationship) that maximizes jump height exist for a given individual’s power capabilities, push-off distance and the angle of push-off^25^. Further research has demonstrated that using the postulated optimal F–V relationship as a guide for prescription of individualized training improves vertical jumping performance^19^. Although improving maximal theoretical power (P_max_) is the most straightforward way to improve vertical jump performance, the results of the aforementioned study showed that individualized training, based on the F–V profile may also contribute to these improvements, without even increasing P_max_. Therefore, assessment of individual’s F–V profile can provide valuable information that can help optimize the strength and conditioning programs.

The traditional multiple-point protocol was shown to be potentially fatiguing^26^, time-consuming and, from the authors practical experience, also potentially dangerous. Building on the fact that F–V relationship is approximately linear and usually very strong^22^, Jaric^15^ and Garcia-Ramos & Jaric^14^ proposed that multiple loads could be replaced with only two loads, without producing significant testing errors (i.e. the two-point method). This was confirmed for several movement tasks, including vertical jumps, cycling, bench press throws, and bench pull27. Recently, Garcia-Ramos and colleagues^26^ tested the two-point method, which involved performing the jump only with zero additional load and with a high load (75 kg). They reported that this protocol is less fatiguing, reliable and valid (compared to the common multiple-point method) for determining F–V profiles for both squat jump (SJ) and counter-movement jump (CMJ). Thus, this approach represents an important optimization of the protocol for F–V profile assessment. While the previous studies have mostly used fixed loads (such as 75 kg in the study by Garcia-Ramos et al.^26^), the validity of the two-point methods that use the high load relative to the individual’s strength ability remains unknown. Although no direct evidence is available on the injury risk of weighted jumping with high loads, very high compressive forces on the lumbar spine (6–10 times body weight) have been reported during half-squats with the loads in the range 0.8 to 1.6 times body weight^28^. Due to ballistic nature of vertical jumps, these compressive loads on the lumbar spine are likely even higher during weighted jumps. In addition, heavy-load resistance exercise has been associated with injury risk^29^, and jumps are known to induce considerable forces on the lower limbs^30^.

The primary aim of this study was to investigate whether the high load within the two-point approach could be replaced with a maximal isometric voluntary contraction (MVC) task, performed in body configuration similar to the one during the push-off phase of jumping. Specifically, we compared the F–V profiles and associated force, velocity and power parameters, obtained by (1) the multiple-point method, (2) the two-point method using bodyweight jumps and high load jumps and (3) a novel Jump-MVC method, using bodyweight jump data and force obtained during an MVC task, performed in a position that resembles the push-off phase. We hypothesized that both the novel Jump-MVC two-point method and the original two-point method^14,15^ are valid approaches for assessing individual’s F–V profile and associated parameters during vertical jumping.

## Methods

### Participants

**Figure 1:**
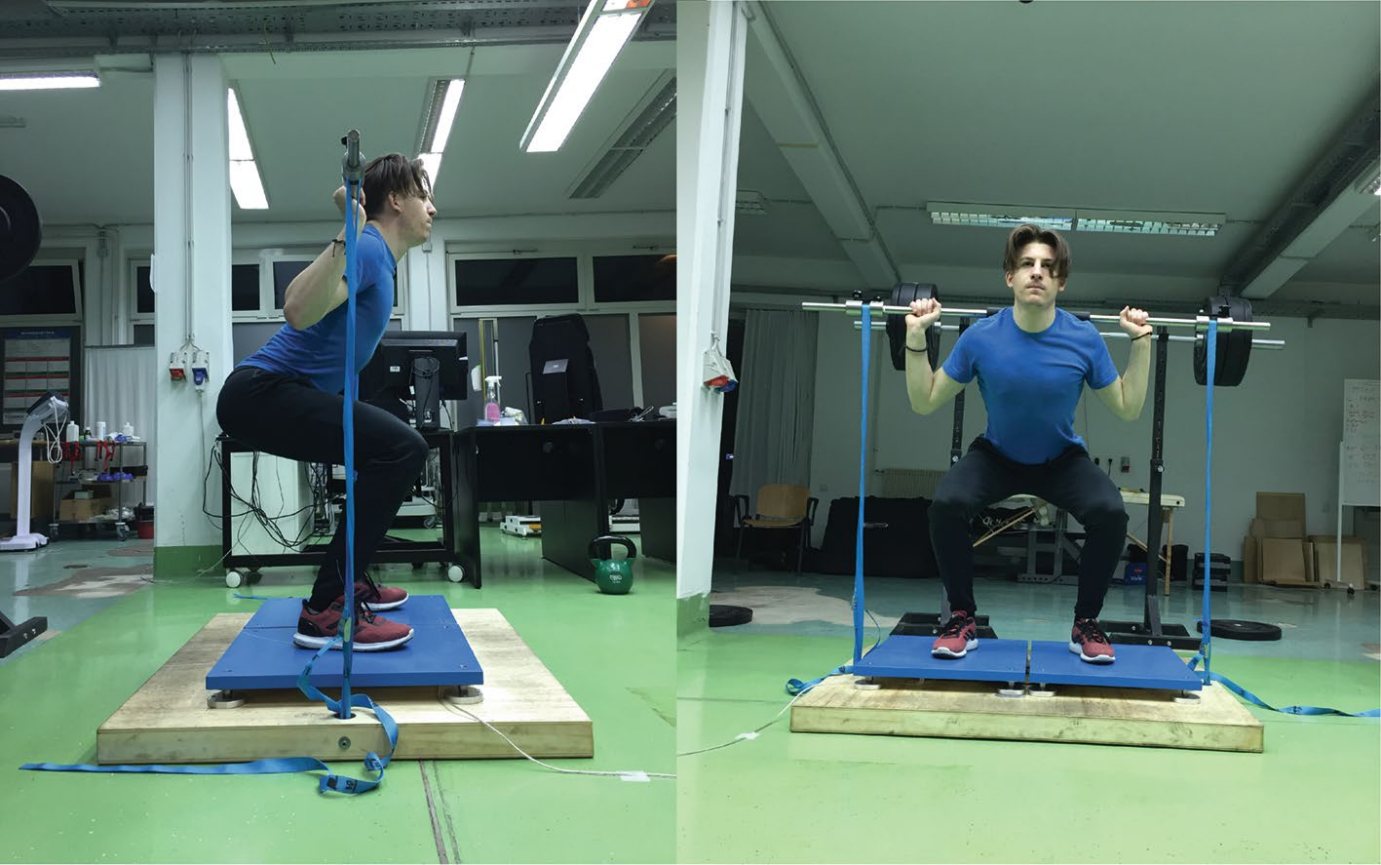
The set-up and positioning during isometric MVC tasks. The length of the bands was adjusted to ensure 30°, 60° and 90° knee angles.

The study population was comprised of 30 young athletes and recreationally active individuals with ≥ 3 years of experience with resistance exercise (21 men; age: 24.2 ± 3.8 years; body height: 180.1 ± 5.9 cm; body mass: 78.8 ± 8.6 kg, 9 women; age: 26.1 ± 2.8 years; body height: 169.6 ± 7.5 cm; body mass: 67.1 ± 10.4 kg). Exclusion criteria were any musculoskeletal injuries and pain syndromes within the previous 12 months and any other health problems that could interfere with the procedure or cause damage to the participants. Participants were thoroughly informed about the protocol in advance and were assured that they may withdraw from the experiment at any point. Informed consent was signed prior to the beginning of the experiment. The protocol was conducted in accordance with the Helsinki declaration and Oviedo convention and was approved by the Republic of Slovenia’s National Medical Ethic Committee (Approval number: 0120–690/2017/8). The participant displayed on Fig. 1 has provided written informed to publish his image alongside the manuscript.

### Study design and procedure

The experiment was conducted in a single session, lasting approximately 90 min. First, the participants performed a warm-up, consisting of 6 min of light aerobic exercise, 13 dynamic stretching exercises (1 set of 6 repetitions each) and 5 bodyweight resistance exercises (1 set of 10 repetitions each). Because not all participants were familiar with the vertical jumping technique, they performed 8 repetitions of SJs and CMJs, with the purpose to familiarize with the task. Technique, but not maximal effort, was emphasized during these introductory jumps.

The main protocol involved performing SJ and CMJ tasks with different loads on a bilateral force plate (Kistler KiJump, Type 9229A, Kistler Instruments, Winterthur, Switzerland). The load was provided with 20-kg Olympic barbell and free weights. Participants always started with zero additional load. In this condition, a lightweight (< 0.5 kg) plastic bar was used instead of the barbell to ensure comparable position to loaded conditions. The next load was set at 20 kg (using only the barbell), and then, the load was gradually increased by adding 10 kg until when and any difficulties in maintaining balance before, during or after the jumps were visually observed by the examiners or if the participants expressed any concerns of continuing, for which they were asked after each load. The protocol was also terminated in case of jump height below 7.5 cm. Three repetitions of SJ and CMJ were performed at each load, and the jump with the maximal achieved height was taken for further analyses. The order of the jump types (SJ and CMJ) were randomized between participant and was kept the same for each participants as the loads increased. The break between repetitions was set at ~ 60 s, and the break between different loads was set at ~ 3 min. Longer breaks were provided at higher loads when participants reported fatigue. The starting position of the SJ^31,32^ and the depth of the countermovement of the CMJ^33^ can heavily influence vertical jump parameters. Therefore, the starting height for SJ and the CMJ depth were recorded and kept constant throughout the trials (the knee angle at 90°). For SJ, an elastic band was positioned for each individual on the appropriate height to be in contact with participants’ buttocks when the desired angle was reached^31,32^. For the CMJ, the elastic band was moved slightly posteriorly relative to the participants to avoid interference and one examiner stood nearby the participant with a straightedge to visually control the countermovement depth.

The session concluded with performing isometric MVC in three positions that closely resembled phases of the push off action, with ground reaction forces being recorded. The positions were determined by the knee angles of 30°, 60° and 90°. The order of positions was randomized between participants. Participants were required to exert maximal force against a bar, tightly strapped to the floor in a way that allowed it to be lifted only to a height corresponding to the respective knee angle value (Fig. 1). Three repetitions, with a 3-s period of maximal exertion, were performed at each knee angle, with 1-min brakes provided between repetitions and between conditions. All participants successfully completed the protocol without any complaints.

### Data processing and outcome measures

Ground reaction force signals were sampled at 1000 Hz and smoothed using a flow arithmetic mean filter (5 ms) as default pre-set by the manufacturer. The trials were captured with the MARS Software (Kistler, Winterthur, Switzerland), which enables immediate and reliable^34^ calculation of the mechanical jump variables. For the purposes of this study, the average force (F_A)_, average velocity (V_A)_ and average power (P_A)_ during the push off phase of the best jump (largest jump height) trial within each condition was used. Based on the F_A_ and V_A_ data across loading condition, the F–V relationship was determined using least squares linear regressions. The intercepts with force axis and velocity axis were calculated to determine the predicted force in isometric conditions (F_0_) and maximal theoretical velocity (V_0_), respectively. The F–v profile was calculated as the slope of the F–v linear relationship, using the following equation^25,26,35^:

$$F{\text{-}}V\, Slope=- \frac{{F}_{0}}{{V}_{0}}$$

Finally, the theoretical maximal power (P_max_) was calculated as in the previous F–V studies^23,26,35,36^, pertaining to jumping performance:

$${P}_{max}=\frac{{F}_{0 }{V}_{0}}{4}$$

For the Jump-MVC method, the same approach was then used to calculate F–V Slope, F_0_, V_0_ and P_max_, by using only the data from jumps with no additional load and isometric MVC in the linear regression. For the MVC task, only force was recorded, and V_A_ was assumed to be zero. These computations were performed using either force data from the best trial for one of the conditions (one knee angle) or the average force of the three MVCs at all three angles. Finally, we calculated the same parameters based only on the bodyweight jump and the highest load for each individual, in order to compare our Jump-MVC method to the original two-point method, as assessed previously by Garcia-Ramos and colleagues^26^, but with high load dictated by individual’s capabilities.

### Statistical analysis

All statistical analyses were performed using SPSS Statistics (Version 20.0. Armonk, NY: IBM Corp.). Descriptive statistics was calculated and reported as mean ± standard deviation. The normality of the data distribution was checked with Shapiro–Wilk test. The differences between the mean values for isometric force among different knee angle conditions was tested with repeated measures analysis of variance and Bonferonni-corrected post-hoc t-tests. The validity of the Jump-MVC and original two-point methods in comparison to the gold standard multiple-point method was tested in several ways. First, we calculated two-way mixed single (ICC_3,1_) and average (ICC_3,k_) intra-class correlation coefficients with respective 95% confidence intervals. The agreement was interpreted as: fair (ICC 0.40–0.59); moderate (ICC 0.60–0.74), and good to excellent (ICC 0.75–1.00)^37^. Second, Bland–Altman plots were also used to further elucidate the agreement between different methods. Third, we used paired-sample t-tests for assessing systematic bias between the methods, and we calculated the standard error of measurement (SEM) and coefficients of variation (CV (%) = SEM/mean × 100) to explore within-individual variation. Finally, ordinary least products (OLP) regression was used to assess bias between the multiple-point and the Jump-MVC method. OLP provides estimates for the intercept and slope of a regression line that accounts for variability in both the x and y axis data. Fixed and proportional bias can be inferred from these estimates using their confidence intervals^38^. Fixed bias is present if the confidence interval of the intercept estimate does not include 0. Proportional bias is present if the confidence interval of the slope estimate does not include 1^38^. In this study, the reported confidence intervals are bias-corrected accelerated confidence intervals derived from bootstrapping the estimates with 10,000 repetitions. The reliability (i.e. the inter-repetition consistency) of MVC trials and single-jump parameters was assessed by ICC, using the same cut-off values as above, and the CV. For all analyses, the outcomes were considered as statistically significant at *p* < 0.05.

## Results

The variables related to the F–V relationship (F_0_, V_0_, P_max_ and F–V slope) were normally distributed for multi-point method (*p* = 0.076–0.770), two-point method (*p* = 0.066–0.601) and for the Jump-MVC method, regardless of the knee angle condition from which the data was used for F–V relationship calculation (*p* = 0.089–0.892).

### Reliability

The reliability (i.e., the inter-repetition consistency) of force measurements during MVC trials was excellent according to the ICC for the knee angles of 30° (ICC = 0.92; 95%CI: 0.80–0.96), 60° (ICC = 0.91; 95%CI: 0.78–0.96) and 90° (ICC = 0.95; 95%CI: 0.92–0.99). Moreover, CV values were acceptable for 30°, 60° and 90° angle (CV = 6.7, 8.3 and 1.6%, respectively).

Similarly, the reliability of jump height was excellent in bodyweight (ICC = 0.97; 95%CI: 0.92–0.99) and loaded conditions (ICC = 0.87–0.96; 95% CI: 0.69–0.99). P_A_ was also analyzed and were also shown as highly reliable for bodyweight jumps (ICC = 0.96; 95% CI: 0.90–0.99) and loaded jumps (ICC = 0.84–0.97; 95% CI: 0.67–0.99). Note that reliability analysis was not done for the 100 kg load, as only 2 participants completed this task (see below for details).

### Jumping ability and strength

There was a substantial between-participant variability of the highest load that the jumps could be performed with (range: 30–100 kg). All participants completed the jumps with at least additional 30 kg. The rest of the loads were achieved by different number of participants as follows: 29 participants up to 40 kg, 25 up to 50 kg, 21 up to 60 kg, 19 up to 70 kg, 10 up to 80 kg, 6 up to 90 kg and 2 up to 100 kg). The average SJ height achieved with the highest load was 10.1 ± 0.3 cm (range = 6.3–15.4 cm). The average CMJ height achieved with the highest load was 11.5 ± 0.4 cm (range = 6.7–17.6 cm).

There was also a high variability between participants in bodyweight jumping performance, with SJ heights in no load condition ranging from 22.5 cm to 42.7 cm (mean value: 31.8 ± 0.07 cm), and CMJ heights in no load condition ranging from 22.1 cm to 47.9 cm (mean value: 34.2 ± 0.07 cm). The force exerted during MVC differed between the three knee angle conditions. The highest mean maximal force was observed at the 30° knee angle (3072.9 ± 631.6 N; range: 1646.6–4204.1 N), followed by the 60° knee angle (2796.9 ± 638.8 N; range: 1593.8–4223.0 N) and the 90° knee angle (1691.3 ± 266.7 N; range: 1178.0–2349.1 N). This difference was statistically significant (F = 185.4; p < 0.001), with pairwise t-tests showing statistically significant differences between all pairs (all p < 0.001).

For the SJ, the F_A_ recorded during the jump with the highest load achieved was 54.8 ± 8.1% of the force during MVC task with 30° knee angle, 60.8 ± 11.2% of the force during MVC with 60° knee angle and 98.2 ± 12.3% of the force during MVC with 90° knee angle. For the CMJ, the F_A_ recorded during the jump with the highest load achieved was 59.2 ± 9.3% of the force during MVC task with 30° knee angle, 65.6 ± 12.7% of the force during MVC with 60° knee angle and 106.2 ± 14.1% of the force during MVC with 90° knee angle.

### Linear force–velocity relationships

For both vertical jumps, the relationship between F_A_ and V_A_ across multiple loads, as assessed by Pearson’s correlation coefficient, was very strong and consistent across participants (SJ: r = − 0.95 ± 0.03, range: 0.84–0.99; CMJ: r = − 0.95 ± 0.04, range: 0.83–0.99).

### Validity of the two-point method

**Figure 2:**
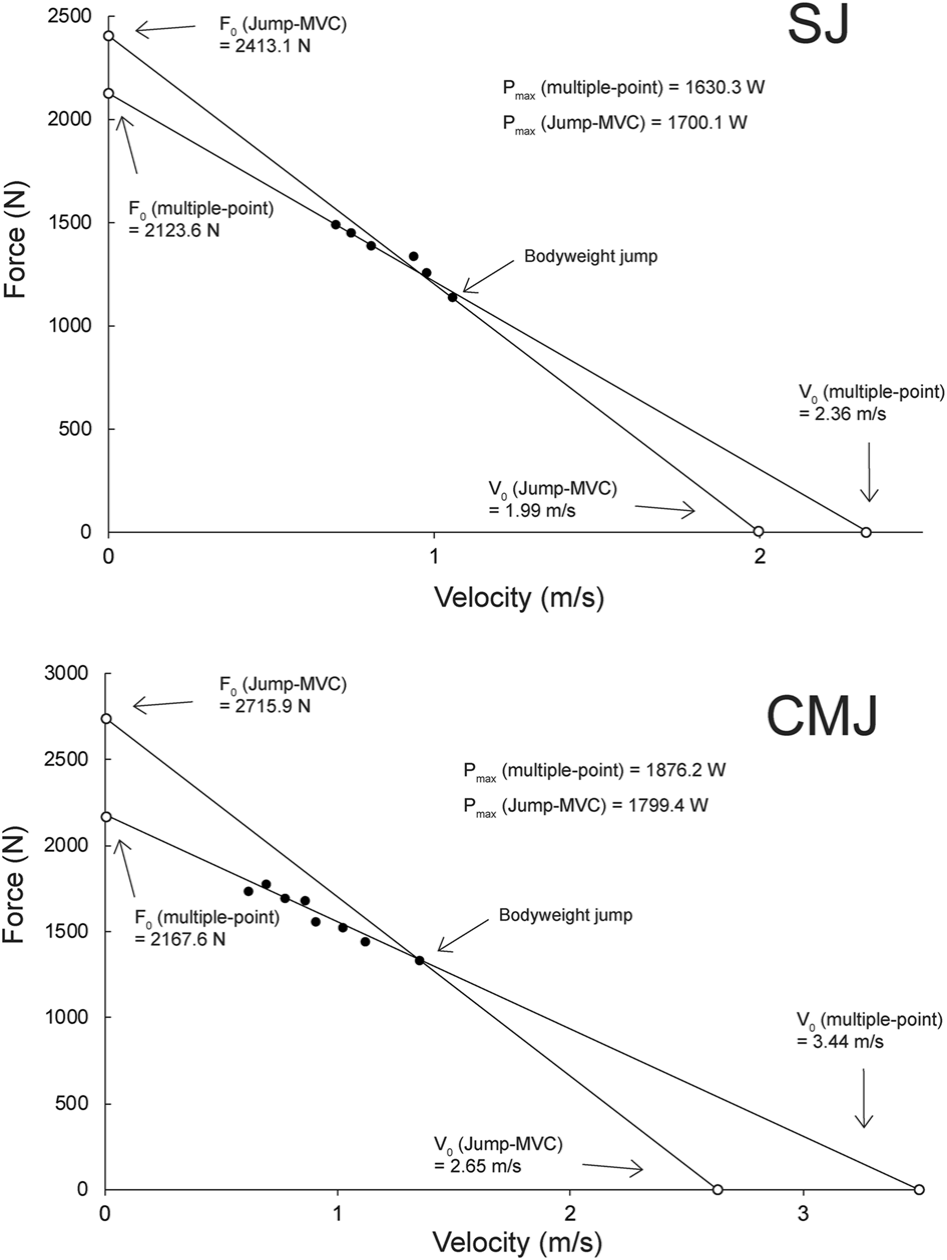
Graphical representation of F–V profiling for a sample subject using multiple-point method and Jump-MVC method, using mean force data from the 30, 60 and 90° angles. SJ—Squat jump, CMJ—countermovement jump, F_0_—maximal force, V_0_—theoretical maximal velocity, P_max_—maximal power.

A comparison of F–V profiles, calculated with the original two-point and with Jump-MVC method for one participant, is shown on Fig. 2. For both SJ and CMJ, the validity of the two-point method was excellent for the slope of the F–V relationship (ICCs = 0.96 and 0.95), and all associated parameters (ICC for F_0_ = 0.97 and 0.98; for V_0_ = 0.98 and 0.91; for P_max_ = 0.99 and 0.97). Detailed results are presented in Table 1.

**Table 1:** Validity of the two-point method for assessment of the force–velocity profile during vertical jumping.

	Mean scores		Intra-class correlation		Within-individual error		Systematic error		
	Multiple-point method	Two-point method	ICCs	ICCa	SEM	CV%	t	Sig	ES
	Squat jump								
F_0_ (N)	2244.2 ± 418.8	2252.7 ± 419.2	0.984 (0.964–0.993)	0.990 (0.982–0.996)	53.6	2.38 (1.86–3.32)	− 0.56	0.582	0.01
V_0_ (m/s)	2.40 ± 0.51	2.37 ± 0.50	0.975 (0.944–0.989)	0.980 (0.971–0.994)	0.1	3.35 (2.62–4.67)	1.03	0.314	0.04
P_max_ (W)	1330.3 ± 304.5	1322.7 ± 307.1	0.991 (0.979–0.996)	0.990 (0.990–0.998)	29.6	2.23 (1.74–3.10)	0.91	0.372	0.03
F–V slope	− 984.0 ± 297.3	− 1001.7 ± 324.6	0.962 (0.917–0.983)	0.980 (0.957–0.991)	60.5	− 6.09 (− 4.76 to − 8.48)	1.03	0.312	0.04
	Counter movement jump								
F_0_ (N)	2193.5 ± 379.4	2206.4 ± 360.4	0.986 (0.968–0.994)	0.990 (0.984–0.997)	44.5	2.02 (1.58–2.81)	− 1.02	0.316	0.04
V_0_ (m/s)	4.50 ± 0.83	4.50 ± 0.83	0.907 (0.800–0.958)	0.950 (0.889–0.979)	0.3	5.73 (4.47–7.97)	− 0.04	0.969	0.00
P_max_ (W)	2450.6 ± 554.3	2484.8 ± 633.4	0.967 (0.927–0.985)	0.980 (0.962–0.993)	107.8	4.37 (3.41–6.077)	− 1.12	0.273	0.05
F–V slope	− 506.1 ± 137.3	− 506.0 ± 128.3	0.949 (0.887–0.977)	0.970 (0.940–0.988)	30.7	− 6.05 (− 4.73 to − 8.43)	− 0.02	0.986	0.00

F_0_–maximal isometric force; V_0_—maximal theoretical velocity; P_max_—maximal power; ICCs—single measures intra-class correlation coefficient; ICCa—average measures intra-class correlation coefficient; SEM—standard error of measurement; CV%—coefficient of variation; ES—Effect size (Cohen’s d).

### Validity of the Jump-MVC method

**Figure 3:**
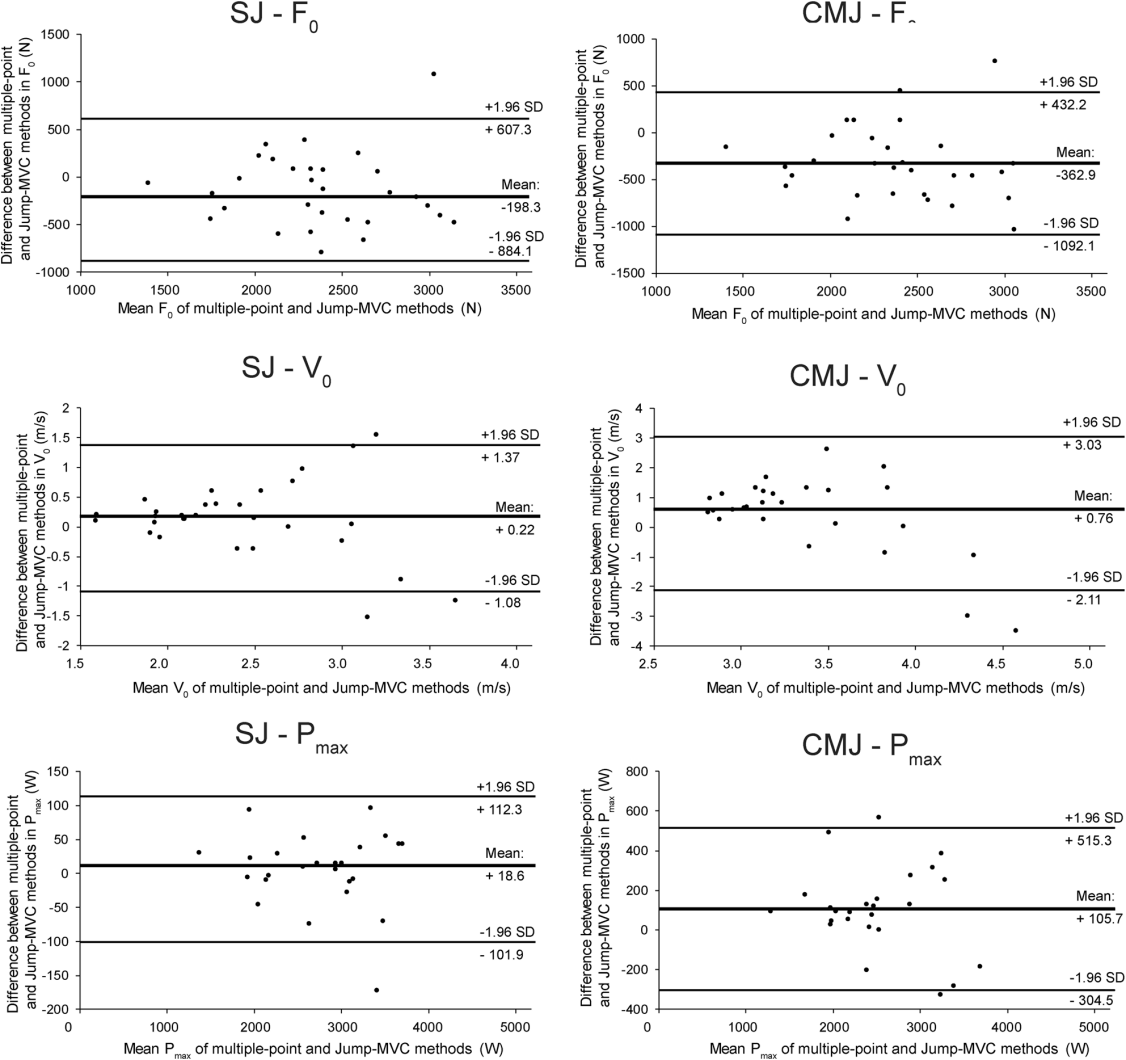
Bland–Altman plots of agreement between multiple-point method and Jump-MVC method, using mean force data from the 30, 60 and 90° angles. SJ—Squat jump, CMJ—countermovement jump, F0—maximal force, V0—theoretical maximal velocity, P_max_—maximal power, SD—standard deviation.

For most parameters associated with F–V profiling, the Jump-MVC method was not in agreement with the multiple-point method when using isometric data from measurements at a single knee angle condition. Using the averaged data across the three angles somewhat improved the agreement, although the validity, particularly the estimations of V_0_ and F–V slope remained poor (Tables 2 and 3). Figure 3 displays Bland–Altman plots for the comparison of multiple-point and Jump-MVC method, using average knee angle data. These results were shown for display as the most reliable (in comparison with single-angle conditions).

**Table 2:** Validity of the Jump-MVC method for assessment of the force–velocity profile in squat jump.

	Mean scores		Intra-class correlation		Within-individual error		Systematic error		
	Multiple-point method	Jump-MVC	ICCs	ICCa	SEM	CV%	t	Sig	ES
	MVC Angle 30°								
F_0_ (N)	2244.2 ± 418.8	2962.4 ± 639.3	0.402 (− 0.093–0.760)	0.570 (− 0.206–0.864)	269.1	10.34 (8.07–14.38)	− 9.44	0.000	0.79
V_0_ (m/s)	2.40 ± 0.51	1.84 ± 0.34	0.365 (− 0.106–0.719)	0.530 (− 0.237–0.837)	0.25	11.75 (9.18–16.35)	7.95	0.000	0.72
P_max_ (W)	1330.3 ± 304.5	1346.6 ± 310.9	0.949 (0.888–0.977)	0.970 (0.941–0.988)	70.2	5.24 (4.095–7.30)	− 0.82	0.418	0.03
F–V slope	− 984.0 ± 297.3	− 1674.6 ± 498.2	0.261 (− 0.089–0.628)	0.410 (− 0.194–0.772)	250.7	− 18.8 (− 14.7 to − 26.2)	9.74	0.000	0.80
	MVC Angle 60°								
F_0_ (N)	2244.2 ± 418.8	2691.8 ± 627.0	0.481 (− 0.035–0.769)	0.640 (− 0.073–0.869)	318.3	12.92 (10.07–17.93)	− 4.97	0.000	0.51
V_0_ (m/s)	2.40 ± 0.51	2.02 ± 0.49	0.440 (0.006–0.723)	0.610 (0.011–0.839)	0.33	15.01 (11.72–20.82)	4.02	0.000	0.40
P_max_ (W)	1330.3 ± 304.5	1329.5 ± 313.1	0.950 (0.890–0.978)	0.970 (0.942–0.989)	70.5	5.3 (4.14–7.38)	0.04	0.970	0.00
F–V slope	− 984.0 ± 297.3	− 1422.8 ± 508.1	0.330 (− 0.087–0.653)	0.490 (− 0.190–0.790)	292.4	− 24.30 (− 18.97 to − 33.81)	5.30	0.000	0.54
	MVC Angle 90°								
F_0_ (N)	2244.2 ± 418.8	1673.3 ± 278.6	0.305 (− 0.085–0.681)	0.460 (− 0.185–0.810)	196.4	10.028 (7.83–13.95)	10.28	0.000	0.81
V_0_ (m/s)	2.40 ± 0.51	4.48 ± 2.51	0.089 (− 0.140–0.374)	0.160 (− 0.326–0.544)	1.68	48.75 (38.062–67.81)	− 4.4	0.000	0.45
P_max_ (W)	1330.3 ± 304.5	1830.2 ± 873.2	0.322 (− 0.040–0.622)	0.480 (− 0.084–0.767)	502.9	31.83 (24.85–44.28)	− 3.51	0.002	0.34
F–V slope	− 984.0 ± 297.3	− 450.3 ± 182.8	0.131 (− 0.074–0.428)	0.230 (− 0.161–0.599)	185.3	− 25.85 (− 20.18 to − 35.96)	− 10.2	0.000	0.81
	MVC Angle: Mean of 30°, 60° and 90° angles								
F_0_ (N)	2244.2 ± 418.8	2442.5 ± 489.8	0.725 (0.349–0.883)	0.840 (0.517–0.938)	210.0	8.96 (7.00–12.47)	− 3.34	0.003	0.32
V_0_ (m/s)	2.40 ± 0.51	2.18 ± 0.50	0.565 (0.218–0.783)	0.720 (0.358–0.878)	0.31	13.76 (10.74–19.14)	2.48	0.021	0.20
P_max_ (W)	1330.3 ± 304.5	1311.7 ± 309.5	0.955 (0.902–0.980)	0.970 (0.949–0.990)	64.9	4.92 (3.84–6.84)	1.01	0.324	0.04
F–V slope	− 984.0 ± 297.3	− 1182.6 ± 363.4	0.570 (0.131–0.803)	0.720 (0.232–0.891)	192.3	− 17.76 (− 13.86 to − 24.70)	3.65	0.001	0.36

F_0_—maximal isometric force; V_0_—maximal theoretical velocity; P_max_—maximal power; MVC—maximal voluntary contraction; ICCs—single measures intra-class correlation coefficient; ICCa—average measures intra-class correlation coefficient; SEM—standard error of measurement; CV%—coefficient of variation; ES—Effect size (Cohen’s d); Note that for the two-point methods, the F_0_ is the actual maximal isometric force (i.e. recorded during maximal voluntary contraction).

**Table 3:** Validity of the Jump-MVC method for assessment of the force–velocity profile in countermovement jump.

	Mean scores		Intra-class correlation		Within-individual error		Systematic error		
	Multiple-point method	Jump-MVC method	ICCs	ICCa	SEM	CV%	t	Sig	ES
	MVC Angle 30°								
F_0_ (N)	2193.5 ± 379.4	3123.3 ± 670.9	0.293 (− 0.075–0.673)	0.450 (− 0.162–0.804)	290.85	10.94 (8.54–15.22)	− 11.30	0.000	0.84
V_0_ (m/s)	4.50 ± 0.83	2.98 ± 0.55	0.177 (− 0.067–0.520)	0.300 (− 0.144–0.684)	0.45	12.16 (9.50–16.92)	11.78	0.000	0.85
P_max_ (W)	2450.6 ± 554.3	2296.1 ± 499.1	0.894 (0.599–0.962)	0.940 (0.749–0.981)	139.27	5.87 (4.58–8.16)	3.93	0.001	0.39
F–V slope	− 506.1 ± 137.3	− 1090.8 ± 336.9	0.133 (− 0.069–0.435)	0.230 (− 0.148–0.606)	186.58	− 23.37 (− 18.25 to − 32.51)	11.08	0.000	0.84
	MVC Angle 60°								
F_0_ (N)	2193.5 ± 379.4	2831.9 ± 680.8	0.361 (− 0.104–0.698)	0.530 (− 0.232–0.822)	349.9	13.93 (10.87–19.38)	− 6.44	0.000	0.63
V_0_ (m/s)	4.50 ± 0.83	3.40 ± 0.97	0.319 (− 0.105–0.659)	0.480 (− 0.234–0.794)	0.61	15.40 (12.024–21.42)	6.38	0.000	0.63
P_max_ (W)	2450.6 ± 554.3	2329.9 ± 565.9	0.911 (0.757–0.964)	0.950 (0.862–0.982)	148.3	6.20 (4.84–8.63)	2.88	0.008	0.26
F–V slope	− 506.1 ± 137.3	− 913.1 ± 372.2	0.180 (− 0.101–0.492)	0.300 (− 0.226–0.660)	223.5	− 31.49 (− 24.59 to − 43.81)	6.44	0.000	0.63
	MVC Angle 90°								
F_0_ (N)	2193.5 ± 379.4	2273.04 ± 468.5	0.721 (0.468–0.866)	0.830 (0.638–0.928)	223.39	10.0 (7.81–13.92)	− 1.26	0.220	0.06
V_0_ (m/s)	4.50 ± 0.83	4.71 ± 1.92	0.426 (0.043–0.700)	0.590 (0.082–0.823)	1.13	24.48 (19.12–34.06)	− 0.65	0.519	0.02
P_max_ (W)	2450.6 ± 554.3	2591.6 ± 906.1	0.762 (0.536–0.887)	0.860 (0.698–0.940)	361.25	14.33 (11.19–19.93)	− 1.38	0.180	0.07
F–V slope	− 506.1 ± 137.3	− 555.9 ± 235.2	0.481 (0.125–0.730)	0.640 (0.222–0.844)	137.99	− 25.98 (− 20.29 to − 36.15)	1.27	0.214	0.06
	MVC Angle: Mean of 30°, 60° and 90° angles								
F_0_ (N)	2193.5 ± 379.4	2556.4 ± 515.4	0.590 (− 0.053–0.848)	0.740 (− 0.111–0.918)	214.67	9.03 (7.05–12.57)	− 5.97	0.000	0.60
V_0_ (m/s)	4.50 ± 0.83	3.74 ± 1.01	0.449 (− 0.021–0.739)	0.620 (− 0.044–0.850)	0.59	14.36 (11.21–19.97)	4.53	0.000	0.46
P_max_ (W)	2450.7 ± 554.3	2345.0 ± 594.3	0.918 (0.797–0.965)	0.950 (0.887–0.982)	150.97	6.30 (4.92–8.76)	2.47	0.021	0.20
F–V slope	− 506.1 ± 137.3	− 734.2 ± 251.8	0.339 (− 0.098–0.671)	0.500 (− 0.217–0.803)	136.51	− 22.01 (− 17.19 to − 30.62)	5.91	0.000	0.59

F_0_—maximal isometric force; V_0_—maximal theoretical velocity; P_max_—maximal power; MVC—maximal voluntary contraction; ICCs—single measures intra-class correlation coefficient; ICCa—average measures intra-class correlation coefficient; SEM—standard error of measurement; CV%—coefficient of variation; ES—Effect size (Cohen’s d); Note that for the two-point methods, the F_0_ is the actual maximal isometric force (i.e. recorded during maximal voluntary contraction).

Specifically, for the SJ, F–V slope estimation was at best fair (ICCs = 0.570) when the mean of the MVC data from all the three knee angle conditions was used. Even using this approach, there was a statistically significant overestimation of the F–V slope (*p* = 0.001). When single knee angle approaches were used, the agreement was poor (ICCs = 0.131–0.330), with larger differences between the mean values. The accuracy of the F_0_ estimation was poor for all of the single knee angle conditions (ICCs = 0.305–0.481) and moderate when data from all angles were combined (ICCs = 0.725). The F_0_ was significantly overestimated for 30° and 60° knee angle conditions, but underestimated for 90° angle (all *p* < 0.001). Similar as for the F_0_, the V_0_ estimation was poor for all the single knee angle conditions (ICCs = 0.089–0.440), and fair when the combination of angles was used (ICCs = 0.565). In contrast to the F_0_, the V_0_ was significantly underestimated for the 30° and the 60° knee angle conditions, but overestimated for the 90° angle (all *p* < 0.001). Estimation of the P_max_ was excellent for all approaches (ICCs = 0.949–0.955), except when 90° knee angle was used (ICCs = 0.322). Detailed results for the SJ trials are presented in Table 2.

Similarly, for the CMJ, F–V slope estimation, using either individual angles or the combination of angles, was poor (ICCs = 0.133–0.481). F_0_ was statistically significantly (all *p* < 0.001) overestimated when data from the 30° knee angle, the 60° knee angle or mean of the three angles was used to compute the F–V relationship, and the estimation was poor to fair (ICCs = 0.293–0.590). If only the data from the 90° knee angle condition was used, there was no statistically significant systematic effect (*p* = 0.220), and the agreement was moderate (ICCs = 0.721). Estimation of V_0_ had poor agreement, regardless of the approach (ICCs = 0.177–0.449), with statistically significant underestimation for all conditions (all *p* < 0.001), except when 90° knee angle data was used (*p* = 0.519). P_max_ estimation had high to excellent agreement for all approaches (ICCs = 0.762–0.918), but there were statistically significant effects for all knee angle conditions (*p*= 0.001–0.021), except for the 90° knee angle (*p* = 0.180). Detailed results for the CMJ trials are presented in Table 3.

**Figure 4:**
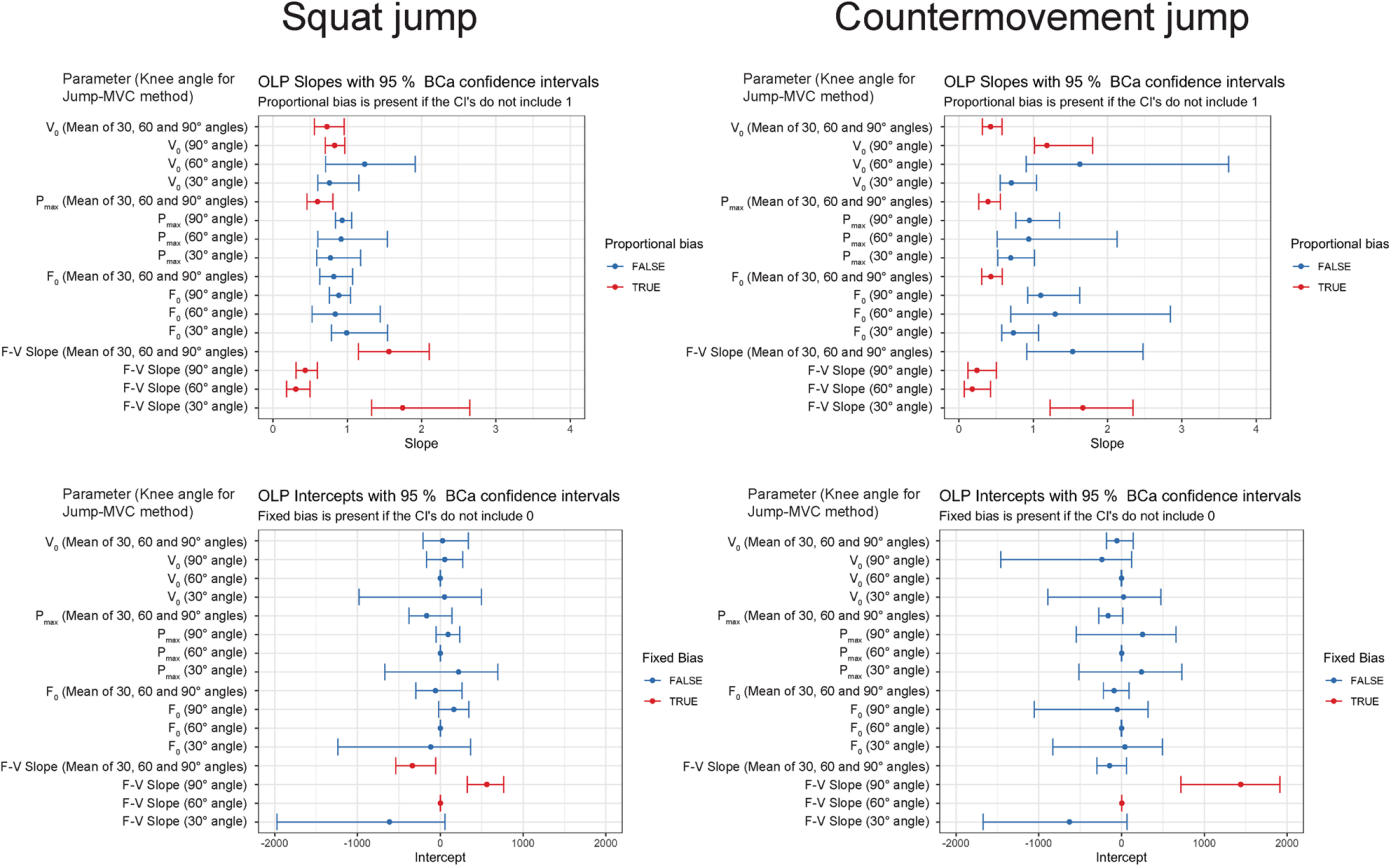
Proportional (upper section) and fixed (lower section) bias between multiple-point and Jump-MVC method, as calculated from the ordinary least product regression (OLP).

According to the OLP regression analysis, proportional bias was present for F–V slope (regardless of the knee angle data for Jump-MVC method) for both jumps (Fig. 4). Moreover, V_0_ was most consistently found to exhibit proportional bias (Fig. 4). For both SJ and CMJ, fixed bias was found only of F–V slopes (Fig. 4).

## Discussion

The purpose of this study was to explore the validity of the novel Jump-MVC method for assessment of jumping F–V profile and associated parameters. In contrast to previous two-point methods, which are based on distal loads, we used a combination of bodyweight jumps and isometric MVC tasks to compute F–V profiles. This novel approach, which used bodyweight jump and isometric MVC, did not prove as a valid method for F–V profiling for jumping tasks, except for the estimation of P_max_. Specifically, most jumping F–V profile parameters, derived using the Jump-MVC method, had poor to fair agreement with the multiple-point method, while also presenting significant systematic and proportional bias. In contrast, excellent validity of slightly modified version of the two-point method that was assessed by Garcia-Ramos and colleagues^27^ was confirmed by our results.

In principle, the linearity of the F–V curve should span from isometric contractions (during which F_0_ is generated) to high velocity movements, such as bodyweight jumping, and likely beyond^16^. The linear F–V relationships for both jumps in our study were strong (r = − 0.95). Some authors have suggested that F–V relationship during single-joint isokinetic tasks might be slightly curvilinear^39^. If this is also the case with multi-joint movements, the overestimation of the maximal isometric force in our study could be partially attributed to non-linearity of the F–V relationship near the zero velocity. However, this is obscured by substantially different force values recorded at different knee angle conditions. Unfortunately, previous studies have rarely used isometric contractions for F–V profiling or comparison of predicted and measured F_0_. There is some evidence that a force plateau is observed when approaching zero velocity in single-joint isokinetic movement^40^, however, this is in contrast with our results and does not explain the inaccuracies of the method we used for F–V profiling. A recent study demonstrated good fit of 1RM (repetition maximum) force during half-squat task to squat jump F–V profile^41^. The 1RM force was lower (~ 5%) than predicted from F–V relationship, but this discrepancy was much smaller than what we observed in our study. However, the 1RM task in the former study did not involve purely isometric conditions (mean vertical velocity: 0.22 m/s). Using 1RM task instead of a jump with high load might therefore be possible instead of using purely isometric MVC. Meanwhile, the reason behind poor agreement between predicted F_0_ and isometric MVC force remains unknown. Possibly, reduced demands for maintaining stability allowed the participants to exert larger forces than they would do in unstable conditions, such as jumping with high loads. It remains unknown whether different methods for isometric MVC measurement would yield different results, for instance, using climbing harness secured to the floor by straps.

It seems that isolating the MVC force recordings to one angle or combination of angles does not provide a reliable estimation of the force capacity through the entire push-off phase, although the validity of the F_0_ estimation was good for SJ if the mean force data from all three angles was used. Perhaps, including more angles and selecting among those would improve the outcomes. Given that F_0_ during SJ was overestimated when MVC was performed with 30° or 60° knee angle, but underestimated when 90° knee angle was used, there is a possibility that an optimal angle exists in-between, at which the isometric force is equal to F_A_ during the push-off phase. If the error of the F_0_ was linearly related to the knee angle used during a MVC task, an angle at which the F_0_ would be accurately estimated should be around 70–75°. It can be speculated that further research could provide an optimal angle to perform the MVC task to maximize the validity of the Jump-MVC method. However, since averaging the data from only 60° and 90° angles yield virtually identical results (results not shown) as combination of all three angles, our data does not support this claim. Estimation of P_max_ was, in contrast to other F–V profile-related parameters, good or excellent for all of the angles used in this study. While this indicates that the novel protocol could be used to assess P_max_, it does not offer any guidance regarding decision to include more force- or velocity-dominant exercise in individual’s training regimen.

The approach to assess F–V profile during vertical jumps with a combination of bodyweight jump and isometric task was influenced and motivated based on previous valid and reliable simplifications of the multiple-point method. In particular, Garcia-Ramos and colleagues^26^ reported high reliability and validity of the two-point method approach using 75 kg load and bodyweight jump. In our study, the validity of similar approach was similar or possibly even higher (ICCs = 0.90–0.99) than the approach in the former study. The differences could have arisen due to somewhat different protocols. In our study, the highest load was different among participants (range: 40–90 kg) in contrast with fixed highest load (75 kg) in the former study^26^. Therefore, participants in our study presumably all performed the last jump at maximal or near-maximal intensity. The average SJ and CMJ height achieved with the highest load was 10.1 ± 0.3 cm and 11.5 ± 0.4 cm in our study. It would be interesting to compare these values with the previous study, however, the authors did not report jump height achieved for the highest load (75 kg). One of the arguments against the approach in our study (i.e., using highest possible load for individuals) is that the vertical jump tests with very heavy loads were reported to have lower reliability compared to bodyweight jumps or moderately loaded jumps^42,43^. Nevertheless, the potentially lower reliability of heavy-loaded jumps did not seem to affect the reliability of F–V profiling in this study. While the modified version of two-point method, used in this study, is possibly more valid than the original two-point method (i.e. using fixed high load), it would probably require additional trials to determine the optimal load for an individual. This would particularly be evident in participants with little experience in performing loaded vertical jumps. In any case, using data from bodyweight jump and loaded jump with sufficiently high load offers a valid method for F–V profiling. On the contrary, replacing high load jump with isometric tasks need further refining and exploration. Previous research that targeted bench press exercise showed that addition of the light loads improved the repeatability of the F–V profiling^44^, which in turn could have an effect on the validity. Therefore, since there was a relatively large gap between bodyweight jump and first loaded jump (20 kg), especially considering that some individuals could only increase the load up to 40 kg (n = 2) or 50 kg (n = 4), perhaps the validity of the multiple-point method as used in this study is also not indisputable. On the other hand, Bland–Altman plots indicate that the largest disagreements between multiple-point and Jump-MVC methods are present for participants with highest F_0_ and V_0_ values.

In addition to improving two-point method with isometric tasks, future research should consider exploring whether the two-point methods could be reliably performed outside laboratory settings (i.e. using jumping mats or smartphone applications that enable calculation of jump height from flight time). It has been confirmed that an alternative multiple-point method (commonly referred to as Samozino’s method), which involves computing the F–V profile using jump heights calculated from flight times, is valid and reliable^36^. Recently, good reliability and validity was also confirmed for the two-point method using this approach^31^. This method currently appears as the best alternative to the standard force plate measurements in terms of time efficiency, fatigue avoidance and safety. The interest of various two-point methods for F–V profiling is rapidly increasing for other movements as well, with important methodological advances recently being made for cycling^35^ and bench press exercise^45^.

Several limitations of the present study should be acknowledged. The sample of participants were comprised of athletes and active individuals. It is possible that the reliability of the two-point methods is population specific. Moreover, while sufficient rest was provided between jumping tasks, it cannot be excluded that fatigue has compromised the performance when the loads were highest (the load was gradually increased in our study). It should be also noted that we did not randomize the order of loads, and we cannot exclude the possibility that this could affect the results of our study. Furthermore, while knee angle was strictly standardized, trunk and ankle position were not. Possibly, more rigorous standardization of the task could affect the outcome of the study. Although the straps were very strongly fixated and never yielded during the experiments, it could have been that the participants were uncomfortable with the MVC task and did not produce their maximal effort. Finally, there was a high between-participant variation in jumping ability, mirrored in bodyweight jump height, as well as the highest load that they performed the jumps with. This could have had an important effect on the outcomes of statistical analyses, as some of the used measures, notably the ICC^46^, are sensitive to the sample heterogeneity. Moreover, many participants were not familiar with vertical jumping testing. Although familiarization trials were provided for both jump types, we cannot exclude the possibility that a certain degree of errors in our study occurred as a consequence non-familiarity with the tasks.

## Conclusion

This study explored the validity of the original and the novel Jump-MVC two-point methods for assessment of F–V profile during jumping. While the excellent validity of a two-point method was confirmed, the novel approach, using bodyweight jump and isometric task proved to have poor to fair validity. Therefore, the two-point method using distal loads is currently the best option for F–V profiling of vertical jumps. Further research should explore different simplifications of the F–V profiling protocols and the feasibility of on-field testing alternatives.

## Consent for publication

The participant displayed on Fig. 1 has provided written informed to publish his image alongside the manuscript.
